# User Interface (UI) Design and User Experience Questionnaire (UEQ) Evaluation of a To-Do List Mobile Application to Support Day-To-Day Life of Older Adults

**DOI:** 10.3390/healthcare10102068

**Published:** 2022-10-18

**Authors:** Di Zhu, Dahua Wang, Ruonan Huang, Yuchen Jing, Li Qiao, Wei Liu

**Affiliations:** 1 School of Design and Architecture, Swinburne University of Technology, Melbourne 3122, Australia; 2 Faculty of Psychology, Beijing Normal University, Beijing 100875, China; 3 Software Technology Center Asia Microsoft, Beijing 518057, China

**Keywords:** user interface design, usability testing, UEQ, mobile application, to-do list, older adults

## Abstract

Because of the spread of smartphones, older adults enjoy the assistance of smartphones. However, fewer mobile applications are designed for older adults. Smartphone user interface (UI) serves as an external brain to capture information, and older adults may have memory complaints that affect self-confidence and lead to memory decline. Non-declarative memory requires more effort. Therefore, this study aims to design and evaluate a to-do list application to help older adults encode, store, and retrieve non-declarative memory, such as tasks they plan to do. We recruited 15 participants (5 men and 10 women) aged 60 to 75 years old (SD = 5.32). They were asked to complete nine usability tasks, and to answer a user experience questionnaire (UEQ) and a few interview questions. Sixty percent of users completed with only one or two attempts (median = 2.80, SD = 1.63). We found three usability issues and proposed an iteration plan. The application has attractiveness, efficiency, dependability, stimulation, novelty, and good perspicuity for older adults. The product was rated excellent except for perspicuity, which met the users’ expectations. This indicates that the user is satisfied with the application prototype. The results of this measurement can be utilized as a benchmark for the next model for developing mobile to-do list applications on user experience.

## 1. Introduction

Older adults report forgetting more frequently and employing memory strategies more regularly. Many older adults have subjective memory complaints that could predict cognitive decline. These results indicated that older persons’ self-reported memory problems might be more closely associated with low mood than with poor memory test performance [1,2]. Moreover, subjective memory complaints may indicate dementia within three years, mainly when objective indicators of memory decline are present [3]. However, a longitudinal study in China’s rural areas showed that subjective memory complaint was related to lower objective memory performance even after researcher-controlled depression and demographic characteristics. Still, subjective memory complaints did not indicate a quicker rate of cognitive decline or dementia throughout three years [4]. Neither depressive symptoms nor well-being was significantly altered by any intervention [5,6]. In other words, older adults with subjective memory complaints and depression are more likely to indicate dementia in three years than older adults without depression. Therefore, this study aims to overcome the memory complaints of older adults without depression.

To overcome the disadvantages of poor memories, two approaches are taken: restorative and compensatory [7]. The restorative approach is the internal development that focuses on maintaining abilities supported by teaching memory techniques to enhance new learning or relearning [7]. Despite variances in training methodologies and participant samples, accumulating data suggest that healthy, non-dementia-afflicted senior citizens can improve and preserve their memory skills. However, traditional methods have not been as successful in proving training transfer, and trainer-led, group-based formats cannot be widely disseminated [8]. Memory training and cognitive stimulation programs may be made more accessible, affordable, and applicable using these innovative techniques, such as information communication technology and high-quality video capture. However, researchers found that training in expectation alteration appeared beneficial in influencing subjective memory complaints. Conventional memory training more effectively influenced objective memory [8]. However, when older adults hold negative stereotypes about aging, they may perform poorly on memory tests and training. Geraci and Miller (2013) attempted to improve older adults’ memory performance by providing them with task experience to counteract their negative performance expectations [9]. Rebok and Balcerak (1989) concluded that memory performance expectations and attributions might influence mnemonic training outcomes [10]. However, self-efficacy and expectation would affect the authenticity of memory test results. Therefore, some older adults do not have specific memory deficits. They need a chance to improve self-efficacy and revise the expectation of memory ability, not improve memory ability. The compensatory approach is external memory aids to support cognitive function and overcome limitations [11]. Researchers introduced the InBath memory aid system for bathroom-related daily care [12], and the fiducial marker tracker (FMT) to check task performance [13]. The findings indicate that assistive technology has the potential to help older adults to live safely and independently in their own homes. The results suggest that a single successful prior task experience can improve older adults’ memory. To do so, the user sets fiducial markers near items photographed by the body-worn camera. However, Schryer and Ross (2013) demonstrated the usefulness of external memory aids. They suggested that older adults may not utilize them to their full potential [14]. As memory aids, we should consider the outdoor use scenario, which contains more privacy concerns and requirements, such as the visible device may refer to the memory problem. However, older adults want to make devices more invisible.

As we mentioned, most older adults may need to correct their expectations of memory performance first and find another way to remember things successfully. The restorative approach aims to improve memory ability directly. However, memory training can be carried out according to the needs of the elderly, but it is not necessary. After all, memory training requires a lot of repetition and time commitment, and the transfer effect is limited. Compared with memory training, or virtual coaching systems, we should merge memory support through mobile application user interfaces (UI) in older adults’ lives, no matter when. The compensatory approach concentrates on memory challenges in daily life. However, it should overcome space limits that expand the scenario out of indoor activities. Therefore, instead of using single approaches, this study combines restorative and compensatory strategies to build confidence in memory ability and reduce the influence on daily activities regardless of time and place. Daily tasks could reflect the memory performance of older adults. Moreover, we should help older adults to set the appropriate expectation of memory ability and provide restorative memory training based on older adults’ needs. Therefore, this study aims to design a mobile to-do list application to support older adults in managing and remembering daily tasks and adjust the expectation of memory performance. Furthermore, this study aims to evaluate whether the user experience of designed prototypes of a mobile to-do list meets user needs. The findings identify the potential areas for improvement, define the level of the user experience, and support further iteration to test memory ability expectation and daily tasks reminding performance.

## 2. Related Work

Research outlines many devices or applications to support older adults, including people with Alzheimer’s disease and Korsakoff syndrome, to overcome perceived memory problems. We summarize the current works to help older adults (see Table 1). Most studies target older adults with memory problems or complaints because of diseases such as Alzheimer’s, Korsakoff syndrome, and chronic illness. These diseases affect participants’ memory ability to remember the people’s names, faces, and things they have done [15,16]. Although these people are valuable to support, we should expand the user group by considering older adults without memory decline. Normal aging older adults are more independent than people with memory decline. However, they still need help under the scenario. Memory has three stages: encoding, storage, and retrieval [17]. Memory problems may begin as early as age 30 and, on average, deteriorate slowly but steadily [18]. For example, older adults struggle to learn about smart TV and remember the new route [19]. Therefore, many digital design solutions aim to support older adults in recording stores and remind activities they have done or need to do.

Considering the feature of digital devices, the devices are good at the store information, including text, images, and video clips. If the device has adequate memory, it can store everything. It is challenging to distinguish the valuable information. Such as the face of people they met and critical moments of daily activities [20]. Unintentional capturing equals encoding information. It relies on the accuracy of the image recognition algorithm. The algorithm defines the values of the image or video clip. Technologies such as wearable cameras and mobile phones could help older adults capture helpful information. Silva et al. (2018) argued that wearable cameras could elicit more than mere familiarity with earlier stimuli and reinstall previous thoughts, feelings, and sensory information, which help older adults to recall [21]. However, it may decrease the self-motivation to memorize the information. The external brain should aim to support the brain rather than replace it. In addition, wearable devices are extra devices in older adults’ daily life, and they may cause a sense of shame. Moreover, to ensure the ability improvement quality, researchers propose a virtual coaching system to help people to execute medical advice or rehabilitation suggestions and improve their self-efficacy by “give people the tools to do something” or “coach” them to learn the skills they need [22]; for example, a virtual coaching system to help people to lose weight and make it easier for them to do so. The system would motivate and guide patients during therapy and help clinicians keep track of how well the therapy is working [23]. However, it lacks merging memory support during everyday activities. Therefore, we should use the technology or device and try to support older adults in daily life, not replace the whole function.

After encoding and storage, we should consider the context of information retrieval. As a memory aids application or device, these should support the information retrieval that provides information when older adults need it. Without a previous setting or active retrieval, it is hard to predict the time to present the information. Therefore, many devices reduce the number of self-initiated processes to avoid missing events [24,25]. Older adults or their caregivers could manage the processes in advance, such as setting a medication calendar or allowing the caregiver to fix it remotely [26]. However, it is time-consuming to search for specific information. In addition, considering the ability of older adults, they are more independent. Many can manage most of the activities. Their children could support them in completing the rest of the activities. However, they would forget to do the activities at the best of times. Therefore, we could solve the challenge by figuring out the best time to remind them so that they have more chances to complete the activities independently.

These solutions serve as external brains for older adults and aim to support their daily activities of older adults. These designs fill the function of the prospective memory gap between normal aging older adults and older adults with memory decline or complaints. Therefore, we recommend the designed application should aim to support storage and provide some clues of encoding and retrieval. With the help of the design, the older adult could give accurate information, recall, and conduct tasks when needed.

## 3. Materials and Methods

### 3.1. Design

This study was conducted in two stages of formative testing. According to the survey by Virzi, four or five participants are enough to find 80% of usability problems [27]. Considering the different smartphone use levels, we recruited 15 participants. In the first stage, we used a cognitive walkthrough based on cognitive theory. The cognitive walkthrough consists of the following steps: receive a goal, search the interface, interact with the interface, and provide feedback [28,29]. UEQ is a semantic difference scale aiming to achieve a quick and direct user experience measurement. A data analytical approach was used to ensure a practical relevance of the constructed scales, i.e., the scales were derived from data concerning a bigger pool of items. Each scale describes a distinct quality aspect of an interactive product [30]. The UEQ scale research team established an evaluation benchmark based on data from many participants assessing 246 products using the UEQ scale, with an average of 40.26 respondents per study. The benchmark consisted of five quality levels: excellent (ranked in the top 10%), good (ranked in the 10–25%), above average (ranked in the 25–50%), below average (ranked in the 50–75%), and poor (ranked in the bottom 25%). We collected verbal feedback and research notes about failure interactions and summarized the opportunities for further iteration. In the second stage, we combined task analysis and the UEQ. We set several tasks for individual participants to complete predefined activities to acquire quantitative data. In addition, we adopted the UEQ as an efficient and reliable way to measure user experience [31].

### 3.2. Participants

A total of 60 participants were recruited through paper and online questionnaires and WeChat groups of the Geriatric psychology laboratory of Beijing Normal University, and 15 older adults were screened by basic information literacy to meet the criteria. The inclusion criteria were (1) older adults aged 60 or above; and (2) have proficiency with smartphones that master at least one digital application. The final sample consisted of 15 participants. Five were men, and ten were women, aged between 60 and 75 years, with a mean age of 65.9 (SD = 5.32). All participants had an education level of junior high school or higher. Among them, middle school education accounted for 20%, high school education accounted for 60%, and college education accounted for 20%.

### 3.3. Materials

#### 3.3.1. Main Software Structure

**Figure 1:**
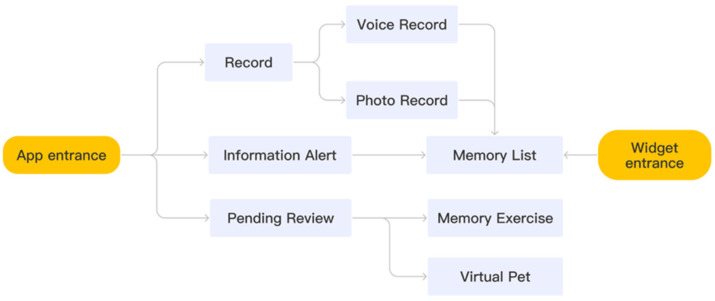
The information structure.

The product has two entrances. One is a regular software entrance where participants can enter the main body of the application by downloading; the other is a widget attached to the software, a shortcut of the application, which displays the reminder content in a conspicuous position on the mobile phone desktop. As Figure 1 shows, the product has three levels.

The essential memory list is used as the product’s home page, exposing critical information, and reducing the path for users to complete the primary behaviour. First, on the main page, the user chooses the way to record data, and the result is displayed on the main page in the form of pictures or texts. The information reminder and to-do review portal are in the page’s upper right corner. After entering the to-do review page, the page enters the second level, which mainly focuses on completing today’s tasks and provides users with other additional function portals, such as a memory competition and achievement system. Older adults could use the application when they have a new task to remember, no matter where or when.

#### 3.3.2. Main Features and Interactions

**Figure 2:**
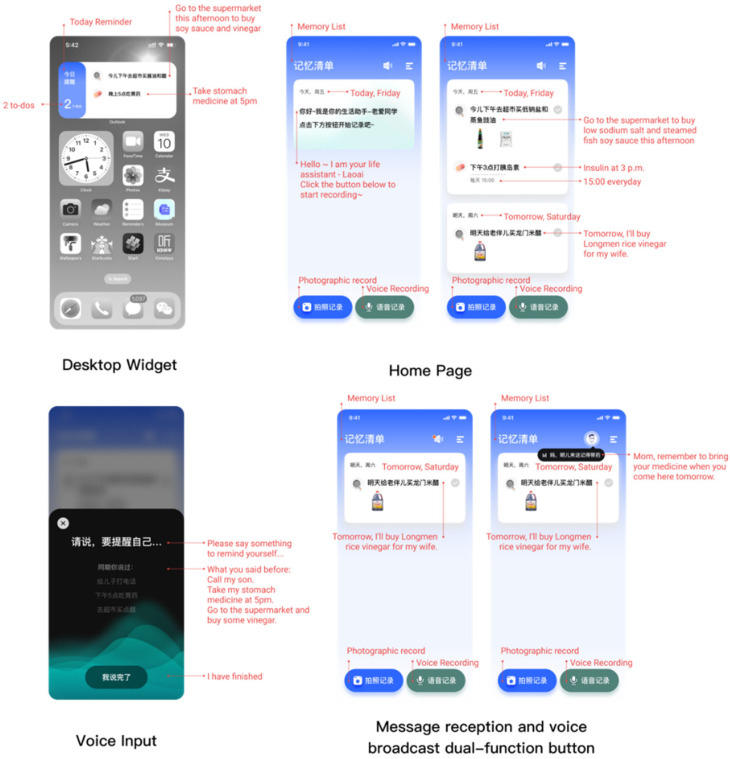

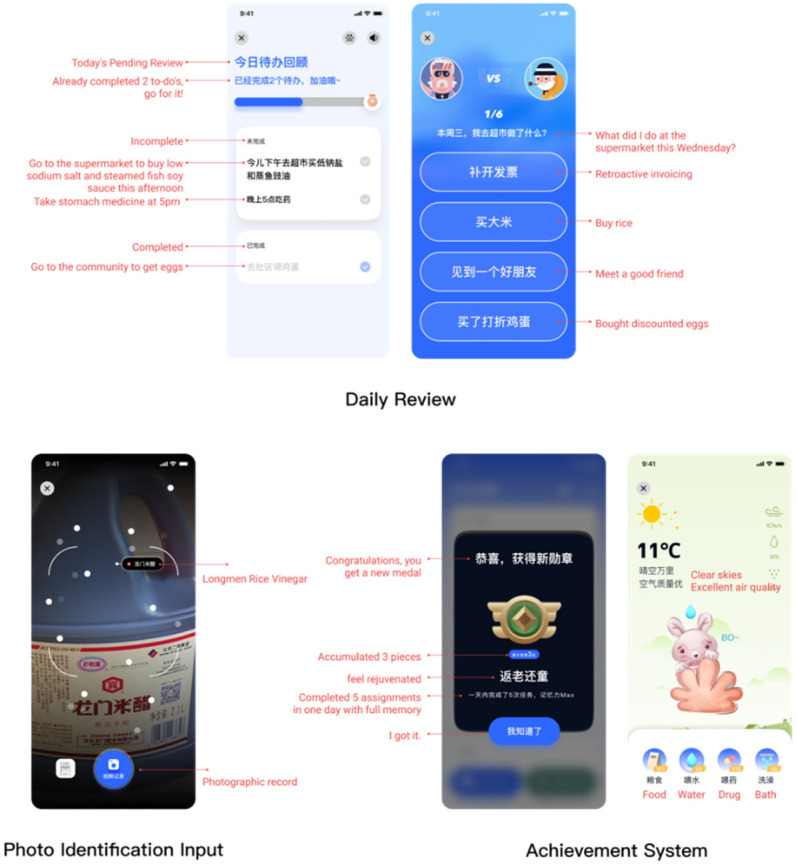
The main features and interactions.

The design goal of this product is to help older adults quickly record and timely remind the trivial life. Specifically, as Figure 2 shows, it includes recording various forms of information anytime, anywhere, timely reminders, and a memory review training system that is highly related to life.

##### Application Icons

The Application Icons allow older adults to check to-do items at any time, thus users can intuitively see unfinished to-do items without entering the application.

##### Home Page

The home page displays a page of the memory list and the entrance of the new mission input feature. The initial interface guides older adults to enter the new mission through active dialogue. Older adults have two forms of input photography and voice. In addition, in the upper right corner of the interface, we designed a dual-function button for reminder and loop broadcast, as well as an entrance to enter the to-do progress. This is different from the 16 pt grid with the left and right distance in the standard Apple iOS specification. We adopted the grid method with the left and right spacing of 20 pt, which can narrow the user’s browsing field of view, so they can focus more on the current display content, and use the single-column card flow of the large-picture layout. The design scheme adopts the single-column card flow form of the large-picture layout. This arrangement consumes the vertical space of the mobile terminal screen. At the same time, it can also help users filter tedious information and only focus on 1–2 contents on one screen.

##### Photo Identification Input

The older adults take pictures to record to-do items, which can improve the efficiency of information entry and, at the same time, ensure the correct rate of information execution of specific tasks, such as purchasing a particular product of a specific brand. After the user points the camera at the item, it automatically retrieves the photographed object and generates a smart tag through image recognition technology. Suppose multiple tags are clicked to take a photo and automatically enter the selection behaviour page. In this case, the feature can quickly generate tasks based on behaviour, object, and time, minimizing the need for input cost. There is a high probability that there is no need to shoot multiple objects continuously, which weakens the secondary shooting.

##### Voice Input

The user records to-do items by voice input. The product automatically recognizes the semantics and displays relevant pictures through the semantics. We designed the dialogue to wait for the other party’s input while adding pre-judgment: when to provide more information, identify additional information from the user, and generate associations with the user’s past. The voice input keeps the conversation moving. At the same time, potential errors are identified and re-interrogated based on more information in the dialogue. By recognizing the user’s voice input information, the content of the dialogue is identified and highlighted. At the same time, to allow more users with difficulty in text recognition to use the product better, the association picture function has been added to confirm their voice input through pictures. These questions and the images come from the presets in advance. When the user has used it for a long time, the photos come from the past shooting records of the older adults.

##### Message Reception and Voice Broadcast

The reminder icon in the header navigation carries different functions in different scenarios. When there is no message, older adults can click to trigger the voice broadcast of the to-do list. When the family collaboration message appears, they click the message bubble to enter the message. At the same time, family members can also enter the to-do items that the older adult needs to do through synchronization and collaboration, record them directly in the list, and present them as a single-column card.

##### Daily Review

Today’s to-do review can query the day’s tasks and present the completion status in a visual form. The completed tasks are greyed out for easy focus and review. When a certain number of tasks are completed within a unit of time, the system automatically triggers the competition mechanism. Users can compete online with users who have had similar tasks in the design and perform retrospective training through recall to improve their prospective memory.

##### Achievement System

After completing a certain number of tasks, the achievement system is triggered, and the corresponding medals are obtained. The product provides a virtual pet companion function, and rewards are accepted according to the degree of achievement completion. The achievement system motivates users to stick to the product and conduct memory review training.

##### Task

According to main features and interactions, we set up 9 tasks, as shown in Table 2. After completing the task, we asked the participants about their feelings, confusion, and preferred approach.

The task was in an offline format, requiring participants to make an audible report during the manipulation process to facilitate an understanding of the motivation behind participants’ behaviour. No direct help was provided when participants encountered difficulties, but prompts were used to guide participants’ thinking to complete the task. All participants tried the prototype in a simulated scenario to complete the test task. After completing the task, the participants were interviewed in a structured manner to understand their true feelings about the product. After completing the task, all participants filled out UEQ scales based on their real-life experiences, recording the strengths and weaknesses of the product for further optimization and iteration.

#### 3.3.3. Variables and Measuring Instruments

As we describe in detail below, the evaluation protocol consists of two parts, namely user information (i.e., collected before the task) and evaluation of task-related information (i.e., performance and users’ opinions). Before conducting usability testing, we collected user profile data. They included demographic data, such as gender and education level. In addition, we assessed users’ information literacy in terms of smartphone usage.

Absolute values determine the success rate: 0 means unsuccessful completion of the task); 1 means successful completion of the task; and 2 means some problems with the task. According to the rules, the experimenter was not allowed to help the participants with the task. However, participants could be given reminders, and they could recall the incarnation’s help, which counted as a new attempt. Any help was annotated. The number of attempts was recorded on the basis that the experimenter had to show the participant how to complete the task.

Most usability scales in Mobile health app testing are designed for expert users, not end-users, such as older adults and caregivers [32]. Compared with typical questions of measuring user experience, UEQ can be answered with relatively low effort, and it offers a convenient way to measure iterations continuously [33]. Therefore, we adopted UEQ, which was suitable for older adults. UEQ evaluates the attractiveness of the product and includes goal-directed quality (i.e., perspicuity, efficiency, dependability) and not goal-directed quality (i.e., stimulation, novelty).

#### 3.3.4. Procedure

Participants were recruited using an online questionnaire, of which 15 met the inclusion criteria and signed a data and records confidentiality agreement. The confidentiality and anonymity of the participants’ data were guaranteed. The experimental session included a usability test in which the experimenter brought all necessary equipment for the study to the experimental site, and participants arrived at the experimental site at the agreed-upon time. First, the experimenter introduced the task and conducted a pre-test assessment protocol (e.g., demographic information and information literacy). After completing the questionnaire, the experimenter explained the task to the participants, showing them the task description through a PowerPoint.

During the test, participants were asked to make an audible report during the manipulation to understand the motivation behind participants’ behaviour. When participants encountered difficulties, instead of providing direct help, they were guided to think about the task by employing prompts. The experimenter encouraged users to complete the task autonomously without giving additional instructions. Success meant the user could complete the task without the experimenter’s help. Attempts were recorded as a failure if any kind of help was needed to complete the task.

For some core interactions, different options were provided for participants to compare and choose from, and participants were asked about their interaction preferences and attitudes toward features. We also asked participants to explain the reasons for their preferences. To obtain reliable data on task performance, we videotaped these sessions. After each task was completed, we conducted structured interviews with participants, asking them about any difficulties or doubts they encountered and how they felt during the task. The sessions were videotaped to obtain reliable data on task performance.

## 4. Results

### 4.1. Main Usability Findings

#### 4.1.1. Finding 1: New Information Reminder Iteration

**Figure 3:**
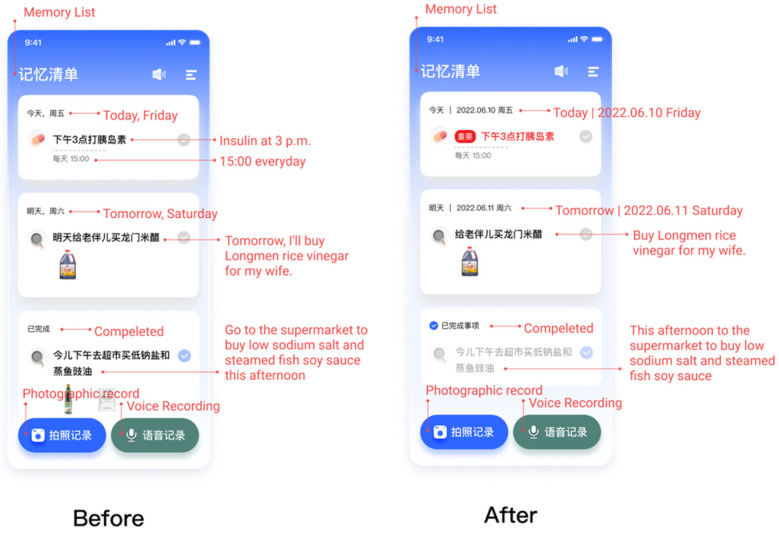
The new information reminder iteration.

A significant problem was the size of the new message display window. Participants could not discover the message display window, which resulted in participants not noticing new message alerts. Another observed usability issue was that participants wanted to display the avatar and name of the message sender when prompted with a new message, so they would know who sent the message on time. To address these issues, as Figure 3 shows, we added names to the avatars so they would see the message sender on time. We expanded the new message alert area, made the small display area more prominent, and added a flashing alert feature.

#### 4.1.2. Finding 2: Attitude toward Achievement System

**Figure 4:**
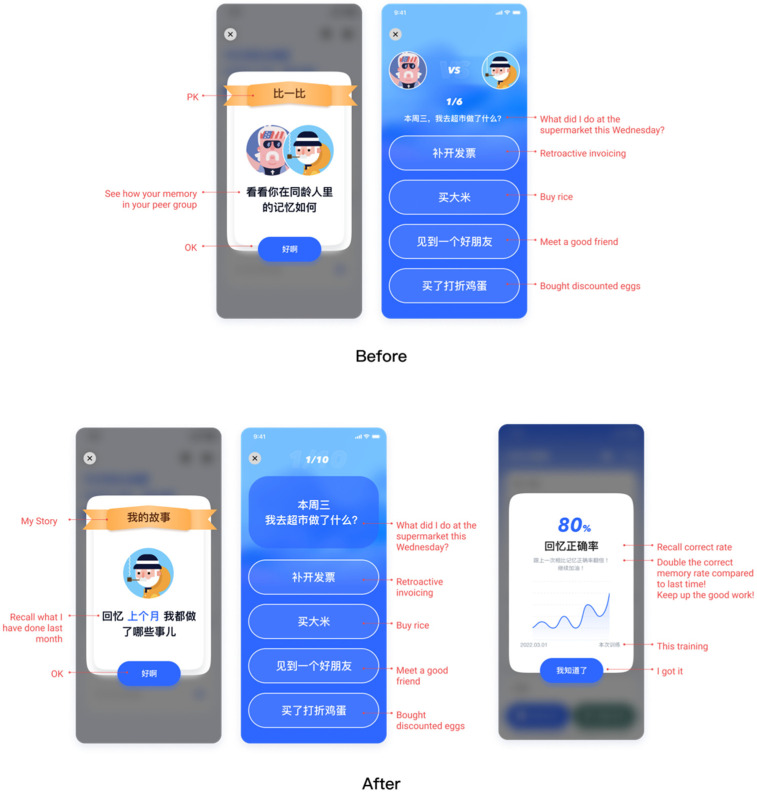
The personal growth chart.

The primary usability findings were related to the participants’ cognitive states and preferences. In the first iteration of usability, most participants indicated that they did not like to compare themselves to others in the PK feature and preferred to focus on their memory. They felt that comparing with others did not have any practical use-value for themselves. Therefore, as Figure 4 shows, we changed the original achievement system of comparing with others to comparing with themselves so that the participants could better focus on their memory condition.

#### 4.1.3. Finding 3: Task Sorting Rules

**Figure 5:**
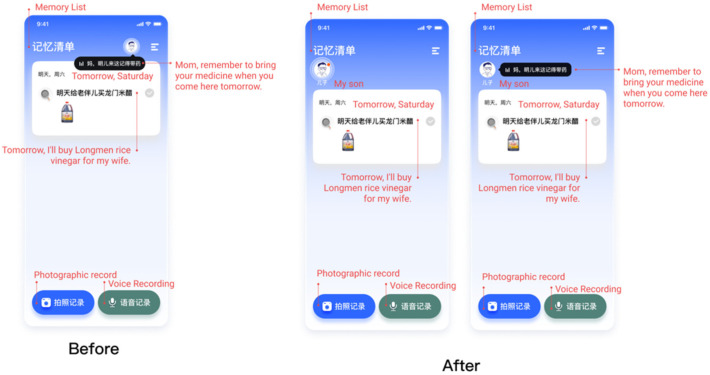
The to-do lists.

One of the usability issues found through observation is the ordering of the to-do list. Many older participants seemed a bit confused when faced with several to-do lists. They did not know which one to perform first and which was more important. At the same time, faced with some completed matters, the participants sometimes wonder whether they have achieved. Therefore, as Figure 5 shows, we iterated the task ordering of the to-do list. All the to-do lists were sorted by time while highlighting important and urgent events, with completed things at the end.

### 4.2. Quantitative Results

The number of attempts to complete the task varied across participants (range 1 to 6). The number of attempts to complete the task varied across participants (range 1 to 6). For comparison, 60% of users completed with only one or two attempts (median = 2.80, SD = 1.63). Comparing the total number of attempts during the study period, we observed that the first two tasks of the study had a lower number of attempts because of the similarity to the way participants interact with WeChat in their daily use.

**Figure 6:**
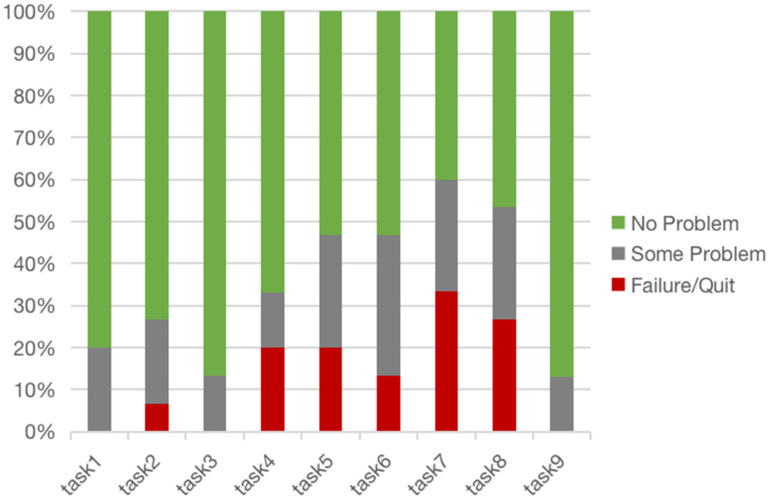
Task success rate.

Based on UEQ scale benchmark from research team, as Table 3 shows, the product’s overall rating is very high, according to the results of the UEQ scale. In their comprehensive evaluation of the product, participants repeatedly mentioned that the product was helpful, meaningful, and needed in real life. However, it was a little challenging to use in some places for the first time, but they could use it well with practice, and they thought the product could improve their memory level. As can be seen from Table 3, the highest mean score was attractiveness with a mean of 2.28 (SD = 0.84), followed by novelty with a mean of 2.10 (SD = 0.90). The ratings were at an excellent level compared to the standard pool, and the subjects considered the product attractive and creative. Most of them expressed a positive attitude toward the product’s effectiveness and were willing to try it. Except for clarity, all other dimensions were in the excellent rank compared to the standard library, mainly due to the lack of experience in using the product for the first time, which led to not understanding the meaning of the page. Figure 6 shows that the success rate of participants in completing the task was recorded during the test.

The completion rates for all tasks were more than 60%, indicating that the task rule descriptions were easy to understand. Task 7 and Task 8 had an incomplete rate higher than 25%, indicating significant problems in the tasks. In Task 7, the main problem of the participants was that they could not find the entrance of the feature and needed more conspicuous access to the feature. In Task 8, editing the memory list, the older adults were unfamiliar with the editing format. They lacked the corresponding experience, making it difficult to understand the meaning expressed on the page.

## 5. Discussion

According to the scores, the product was rated as excellent except for perspicuity, which met the users’ expectations. According to the question descriptions and the scale scores, the participants thought the product design was evident at a glance but not easy to understand, challenging to learn, and somewhat complicated. The reasons for this were that some of the product’s features were not transferable to the participants because they had no previous experience with the product and required some practice and adaptation time. Older adults have different interaction preferences than younger groups because of aging barriers [34]. The older adults’ acceptance of interaction is related to their previous experience. Participants have a higher success rate on voice input than we expected. Because the interaction of the voice input feature is similar to WeChat: a long press of the button for voice input, this phenomenon is also related to the popularity of WeChat in China [35].

Moreover, the desktop Widget is a new concept for older adults. Older adults hold a positive attitude toward the widget. However, most of them have never used it before. We need to test the performance of the interaction. To reduce the information space occupied by the home page, the reminder icon button in the header navigation is set in the form of a double function button, i.e., the icon carries different functions in different scenarios. When there is no message, you can trigger the voice broadcast of the to-do content by clicking on it. When the family collaboration message appears, the message is recorded into the to-do list by clicking on the message bubble. Although the interface design criteria emphasize consistency [36], considering the limited information older adults can process, different information share the same location. Older adults may feel confused with the same location of two different information. However, they could not perceive the inconsistency between the same location and two types of information. Therefore, we should further investigate how older adults interact with dual functions when they get familiar with the product. Therefore, to further explore the interaction modes suitable for the older adults, we should also consider the differences in learning costs of various interaction modes after excluding the influence of previous experience and investigate whether older adults could customize the desktop of the mobile phone.

The participants reported that grocery shopping is widespread by using a product scenario. If they only buy one thing, they prefer to carry the sample, such as an empty bottle. We should focus on the products that are hard to maintain or easy to lose, such as many medicine bottles and necessary receipts. In addition, except for daily tasks, we should focus more on non-declarative memory techniques that older adults can master after practicing [37]. Older adults could find it difficult when they memorize the sequencing tasks. Therefore, the memory of the action to use the application is easier for them. They can decide when to use the product from cues from the external brain, save the latest medication prescription, or a new recipe.

Older adults have a positive attitude toward comparing with previous cognitive testing results and are unwilling to compare with others. Older adults recognize the necessity of screening for diseases such as Alzheimer’s [38]. In addition, they focus more on effectiveness and usefulness. Older adults said, an “achievement system is cute but not useful”. Kappen et al. (2016) found that gamification elements such as leaderboard and achievement systems may not be suitable for older adults [39]. They suggested letting older adults reward each other. However, we did not identify the reason behind the attitude. We could not determine a better plan. Older adults may want to save energy for more important activities, or they may be afraid of failure. Therefore, we prefer to present self-growth charts and provide regular cognitive tests. In the future, we will consider techniques for learning memory to help older adults reduce memory burden and save energy in learning other techniques more easily [40,41,42].

## 6. Conclusions

This paper presents the UI design and UEQ evaluation of external memory aids for older adults. The application has excellent attractiveness, efficiency, dependability, stimulation, novelty, and good perspicuity for older adults. Acting as an external brain of older adults, it follows the encoding, storage, and retrieval stages. The encoding stage provides potential options and information categories to stimulate older adults to specify the task. At the storage stage, the task ranks by the time, and shows the most urgent tasks on the top of the task list. At the retrieval stage, the product give reminders to older adults that are activated by the time or location. In the future, we plan to iterate the product with a note feature that supports saving important information. In addition, when designing digital products for older adults, we should consider their previous technology usage and encourage their motivation to use products by tracking their performance. Though the developed application had limited functions, it is an example of a digital application proposing a productive application for older adults. However, the designed application is a mock-up that requires further effectiveness validation [43,44,45]. We plan to iterate, implement, and test UI designs with a functional demo that supports effectiveness evaluation.

## Figures and Tables

**Table 1:** Summary of existing memory aids for older adults.

Project	Author	Platform	User	Feature	Advantage	Disadvantage
SenseCam	(A. R. Silva et al., 2017)	Wearable Cameras, PC	AD patients	Maximum collection of images from everyday life (moments/events that can remain private), comments on the images viewed	Allows users to recall memories by automatically recording past time	Requires medical staff assistance. Regular reviews need large amounts of storage space and computing power. Potential privacy issues with long-term wear.
FMT	(Li et al., 2019)	Wearable cameras, cell phones	Experience with smartphones, 65+ years old	Automatically captures images based on markers near the tracking object set by the user in life	Record the critical points of daily life and save a lot of storage space on the device	Additional help is needed to install the markers. Adds the burden of carrying extra devices to the user daily. There is a lot of information and searching and finding specific objects is tricky.
MyWepp	(Lloyd, Oudman, Altgassen, & Postma, 2019)	Smartwatch	KS Patients	Task reminders and clocks	Better at completing accurate and timely PM tasks, allowing caregivers to control remotely	Additional wearable devices are uncomfortable to wear and require some cognitive ability to learn to use the management system independently.
SSA	(McCarron et al., 2019)	Cell phones and smartwatches	Dementia or mild cognitive impairment/self-identified memory problems	Facial Recognition	Helps disaffected people remember the names and relationships of the people they interact with	Complex registration and login process. Interaction in daily life is not natural. Wearing additional assistive devices tends to create a sense of stigma
Smartphone Calendar	(El Haj, Moustafa, Gallouj, and Allain, 2021)	Smartphone	Patients with mild AD	Add events	The reduced self-initiation process results in fewer missed events	Technical and experience limitations.
Medisafe	(Chaudhry, 2016)	Smartphone	Patients who require long-term medication	Medication management, medication reminders, dosing reports	Record and remind time to take medication Generate reminder reports Simplify medication administration details Doctor Appointment Reminder	Lack of initiative to motivate users. Difficult to adhere to.

**Table 2:** The task List.

No.	Task	Process	Interviews
1	Remind yourself to “buy non-iodized salt and soy sauce this afternoon” by voice recording.	Successfully entered and set reminders.	What do you think of the virtual pet feature? Why? What is your preferred way of sorting tasks? (Time-division? Do they complete tasks last? Time-division? Have completed tasks not shown? (Another arrangement?) What kind of message display do you prefer? (Show details? Text only? Show thumbnails? Other display methods?) How do you prefer to view message alerts? (All tucked away and viewed in one place? Show all family members’ avatars?)
2	Remind yourself to “take insulin every day at 3 pm” by voice recording.	Enter error, re-enter.	
3	Remind yourself to “remember to buy Longmen rice vinegar for your partner tomorrow” by taking photos.	Successfully entered and set reminders.	
4	Dealing with the completed tasks 1. go to the supermarket this afternoon to buy low sodium salt and steamed fish and soy sauce 2. take medicine at 5 pm	Check the list of completed memories.	
5	View reminders sent by your son and add them as new tasks.	View notifications and one-click settings.	
6	Perform memory review exercises.	Memory PK feature.	
7	Please feed your pet.	Check your pet’s status.	
8	Edit the memory list.	Complete the edit and save.	
9	View reminders 2.	Successfully view reminders 2.	

**Table 3:** The UEQ Result.

Dimension	Mean	Variance	Rating
Attractiveness	2.28	0.84	Excellent
Perspicuity	1.78	1.08	Good
Efficiency	2.10	0.90	Excellent
Dependability	1.87	1.23	Excellent
Stimulation	2.05	1.04	Excellent
Novelty	1.95	1.30	Excellent

## Data Availability

Not applicable.

## References

[1] Al Mahmud A., Long K.M., Harrington K.D., Casey K., Bhar S., Curran S., Hunter K., Lim M.H. (2022). Developing a digital psychoeducational tool to reduce loneliness in older adults: A design case study. Int. J. Hum. Comput. Interact..

[2] Bolla K.I., Lindgren K.N., Bonaccorsy C., Bleecker M.L. (1991). Memory complaints in older adults: Fact or fiction?. Arch. Neurol..

[3] Schmand B., Jonker C., Hooijer C., Lindeboom J. (1996). Subjective memory complaints may announce dementia. Neurology.

[4] Wang P.N., Wang S.J., Fuh J.L., Teng E.L., Liu C.Y., Lin C.H., Shyu H.Y., Lu S.R., Chen C.C., Liu H.C. (2000). Subjective memory complaint in relation to cognitive performance and depression: A longitudinal study of a rural Chinese population. J. Am. Geriatr. Soc..

[5] Metternich B., Kosch D., Kriston L., Härter M., Hüll M. (2010). The effects of nonpharmacological interventions on subjective memory complaints: A systematic review and meta-analysis. Psychother. Psychosom..

[6] Tang M., Wang D., Guerrien A. (2020). A systematic review and meta-analysis on basic psychological need satisfaction, motivation, and well-being in later life: Contributions of self-determination theory. PsyCh J..

[7] Huckans M., Hutson L., Twamley E., Jak A., Kaye J., Storzbach D. (2013). Efficacy of cognitive rehabilitation therapies for mild cognitive impairment (MCI) in older adults: Working toward a theoretical model and evidence-based interventions. Neuropsychol. Rev..

[8] Gross A.L., Parisi J.M., Spira A.P., Kueider A.M., Ko J.Y., Saczynski J.S., Samus Q.M., Rebok G.W. (2012). Memory training interventions for older adults: A meta-analysis. Aging Ment. Health.

[9] Geraci L., Miller T.M. (2013). Improving older adults’ memory performance using prior task success. Psychol. Aging.

[10] Rebok G.W., Balcerak L.J. (1989). Memory self-efficacy and performance differences in young and old adults: The effect of mnemonic training. Dev. Psychol..

[11] Morrow-Howell N. (2010). Volunteering in later life: Research frontiers. J. Gerontol. Ser. B.

[12] Bayen U.J., Dogangün A., Grundgeiger T., Haese A., Stockmanns G., Ziegler J. (2013). Evaluating the effectiveness of a memory aid system. Gerontology.

[13] Li F.M., Chen D.L., Fan M., Truong K.N. FMT: A wearable camera-based object tracking memory aid for older adults. Proceedings of the ACM on Interactive Mobile, Wearable and Ubiquitous Technologies.

[14] Schryer E., Ross M. (2013). The use and benefits of external memory aids in older and younger adults. Appl. Cogn. Psychol..

[15] McCarron H.R., Zmora R., Gaugler J.E. (2019). A web-based mobile app with a smartwatch to support social engagement in persons with memory loss: Pilot randomized controlled trial. JMIR Aging.

[16] Silva A.R., Pinho M.S., Macedo L., Moulin C., Caldeira S., Firmino H. (2017). It is not only memory: Effects of sensecam on improving well-being in patients with mild Alzheimer disease. Int. Psychogeriatr..

[17] McDermott K.B., Roediger H.L. (2018). Memory (Encoding, Storage, Retrieval).

[18] Christensen H. (2001). What cognitive changes can be expected with normal ageing?. Aust. N. Z. J. Psychiatry.

[19] Gitlow L. (2014). Technology use by older adults and barriers to using technology. Phys. Occup. Ther. Geriatr..

[20] Lloyd B., Oudman E., Altgassen M., Postma A. (2019). Smartwatch aids time-based prospective memory in Korsakoff syndrome: A case study. Neurocase.

[21] Silva A., Pinho M., Macedo L., Moulin C. (2018). A critical review of the effects of wearable cameras on memory. Neuropsychol. Rehabil..

[22] Demrozi F., Serlonghi N., Turetta C., Pravadelli C., Pravadelli G. Exploiting Bluetooth low energy smart tags for virtual coaching. Proceedings of the 2021 IEEE 7th World Forum on Internet of Things (WF-IoT).

[23] Bissoli L., Bonacina D., Dalla Riva N., Demrozi F., Jereghi M., Marchiotto N., Perbellini G., Pernice B., Pizzocaro E., Pravadelli G. A virtual coaching platform to support therapy compliance in obesity. Proceedings of the IEEE 46th Annual Computers, Software, and Applications Conference.

[24] Chaudhry B.M. (2016). Health is fine if pills are on time. mHealth.

[25] Zhu D., Zhang B., Wu J., Zhao L., Jing Y., Wang D., Liu W., Al Mahmud A., Qiao L., Auernhammer J. Social inclusion in an aging world: Envisioning elderly-friendly digital interfaces. Proceedings of the 5th International Conference on Human Interaction and Emerging Technologies.

[26] El Haj M., Moustafa A.A., Gallouj K., Allain P. (2021). Cuing prospective memory with smartphone-based calendars in Alzheimer’s disease. Arch. Clin. Neuropsychol..

[27] Virzi R.A. (1992). Refining the test phase of usability evaluation: How many subjects is enough?. Hum. Factors.

[28] Desmet P., Xue H., Xin X., Liu W. Emotion deep dive for designers: Seven propositions that operationalize emotions in design innovation. Proceedings of the International Conference on Applied Human Factors and Ergonomics (AHFE International).

[29] Rieman J., Franzke M., Redmiles D. Usability evaluation with the cognitive walkthrough. Proceedings of the Conference Companion on Human Factors in Computing Systems.

[30] Schrepp M., Hinderks A., Thomaschewski J. Applying the user experience questionnaire (UEQ) in different evaluation scenarios. Proceedings of the International Conference of Design, User Experience, and Usability, Heraklion.

[31] Azad-Khaneghah P., Neubauer N., Miguel Cruz A., Liu L. (2021). Mobile health app usability and quality rating scales: A systematic review. Disabil. Rehabil. Assist. Technol..

[32] Schrepp M. Measuring user experience with modular questionnaires. Proceedings of the International Conference on Advanced Computer Science and Information Systems.

[33] Schrepp M., Thomaschewski J., Hinderks A. (2017). Construction of a benchmark for the user experience questionnaire (UEQ). Int. J. Interact. Multimedia Artif. Intell..

[34] Wildenbos G.A., Jaspers M.W., Schijven M.P., Dusseljee-Peute L. (2019). Mobile health for older adult patients: Using an aging barriers framework to classify usability problems. Int. J. Med. Inform..

[35] Song L., Ge Y., Zhang X. (2021). The relationship between WeChat use by Chinese urban older adults living alone and their subjective well-being: The mediation role of intergenerational support and social activity. Psychol. Res. Behav. Manag..

[36] Nielsen J., Molich R. Heuristic evaluation of user interfaces. Proceedings of the SIGCHI Conference on Human Factors in Computing Systems.

[37] Woodruff-Pak D.S., Finkbiner R.G. (1995). Larger nondeclarative than declarative deficits in learning and memory in human aging. Psychol. Aging.

[38] Braun S.R., Reiner K., Tegeler C., Bucholtz N., Boustani M.A., Steinhagen-Thiessen E. (2014). Acceptance of and attitudes towards Alzheimer’s disease screening in elderly German adults. Int. Psychogeriatr..

[39] Kappen D.L., Nacke L.E., Gerling K.M., Tsotsos L.E. Design strategies for gamified physical activity applications for older adults. Proceedings of the 49th Hawaii International Conference on System Sciences.

[40] Flach J.M., Stappers P.J., Voorhorst F.A. (2017). Beyond affordances: Closing the generalization gap between design and cognitive science. Des. Issues.

[41] Gray C.M. (2022). Languaging design methods. Des. Stud..

[42] Buur J., Ankenbrand B., Mitchell R. (2013). Participatory business modelling. CoDesign.

[43] Desmet P., Fokkinga S. (2020). Beyond Maslow’s pyramid: Introducing a typology of thirteen fundamental needs for human-centered design. Multimodal Technol. Interact..

[44] Peters D., Ahmadpour N., Calvo R.A. (2020). Tools for wellbeing-supportive design: Features, characteristics, and prototypes. Multimodal Technol. Interact..

[45] Gough P., Yoo S., Tomitsch M., Ahmadpour N. (2021). Applying bioaffordances through an inquiry-based model: A literature review of interactive biodesign. Int. J. Hum. Comput. Interact..

